# A population-based survey of prevalence of diabetes and correlates in an urban slum community in Nairobi, Kenya

**DOI:** 10.1186/1471-2458-13-371

**Published:** 2013-04-20

**Authors:** Richard Ayah, Mark D Joshi, Rosemary Wanjiru, Elijah K Njau, C Fredrick Otieno, Erastus K Njeru, Kenneth K Mutai

**Affiliations:** 1 School of Public Health, College of Health Sciences, University of Nairobi, P.O. BOX 19676–00202, KNH, Nairobi, Kenya; 2 School of Medicine, College of Health Sciences, University of Nairobi, Nairobi, Kenya; 3 University of Nairobi Partnership for Advanced Care and Treatment (PACT), Centre of Excellence, Nairobi, Kenya

**Keywords:** Diabetes mellitus/epidemiology, Urban slum, Sub-Saharan Africa, Diabetes correlates, Epidemiological transition

## Abstract

**Background:**

Urban slum populations in Africa continue to grow faster than national populations. Health strategies that focus on non-communicable diseases (NCD) in this segment of the population are generally lacking. We determined the prevalence of diabetes and associated cardiovascular disease (CVD) risk factors correlates in Kibera, Nairobi’s largest slum.

**Methods:**

We conducted a population-based household survey utilising cluster sampling with probability proportional to size. Households were selected using a random walk method and consenting residents aged 18 years and above were recruited. The WHO STEPS instrument was administered. A random capillary blood sugar (RCBS) was obtained; known persons with diabetes and subjects with a RCBS >11.1 had an 8 hours fasting blood sugar (FBS) drawn. Diabetes was defined as a RCBS of ≥ 11.1 mmol/l and a FBS of ≥ 7.0 mmol/l, or a prior diagnosis or receiving diabetes drug treatment.

**Results:**

Out of 2061 enrolled; 50.9% were males, mean age was 33.4 years and 87% had a minimum of primary education. Only 10.6% had ever had a blood sugar measurement. Age adjusted prevalence of diabetes was 5.3% (95% CI 4.2-6.4) and prevalence increased with age peaking at 10.5% (95% CI 6.8-14.3%) in the 45–54 year age category. Diabetes mellitus (DM) correlates were: 13.1% smoking, 74.9% alcohol consumption, 75.7% high level of physical activity; 16.3% obese and 29% overweight with higher rates in women.

Among persons with diabetes the odds of obesity, elevated waist circumference and hypertension were three, two and three fold respectively compared to those without diabetes. Cardiovascular risk factors among subjects with diabetes were high and mirrored that of the entire sample; however they had a significantly higher use of tobacco.

**Conclusions:**

This previously unstudied urban slum has a high prevalence of DM yet low screening rates. Key correlates include cigarette smoking and high alcohol consumption. However high levels of physical activity were also reported. Findings have important implications for NCD prevention and care. For this rapidly growing youthful urban slum population policy makers need to focus their attention on strategies that address not just communicable diseases but non communicable diseases as well.

## Background

The global prevalence of non-communicable diseases (NCD) dominated by diabetes [1] is increasing with the greatest burden occurring in developing countries, and it is projected that by the year 2020, NCD’s will surpass communicable diseases as a cause of death [2,3]. Current disease estimates for Sub-Saharan Africa (SSA) are based on sparse data, but projections indicate increases in NCD’s caused by demographic and epidemiologic transitions.

The world prevalence of diabetes among adults was projected at 6.4% in 2010, affecting 285 million adults, and by 2030, it is estimated to be 7.7% with 439 million adults affected [4]. Much of this burden will be in developing countries with an increase of 69% in the number of adults with diabetes [1]. The urban poor are the population segment most likely to be affected. Low socio-economic status including low education levels has been associated with development of diabetes mellitus [5,6]. An association between increasing poverty levels and increasing prevalence of diabetes has been shown among women but is not as strong for men. [7,8]. A study in India among a poor urban slum population generally believed to have low risk for lifestyle diseases found a high prevalence of risk factors for NCD’s [9].

Currently the estimated prevalence of diabetes in Africa is 1–3% in rural areas and 5-6% in urban Sub Saharan Africa however country reports have varied widely [10]. The International Diabetes Federation Atlas estimated that in 2006, 10.8 million people had diabetes in Sub Saharan Africa and that this would increase to 18.7 million by 2025, an increase of 80% exceeding the predicted worldwide increase of 55% [11]. The epidemiology of diabetes in Kenya has not been studied to any great extent. Anecdotal evidence from health care services suggests that the incidence of diabetes is on the increase. The best estimate of diabetes is from an opportunity sample of an urban and rural population that reported a non-age adjusted prevalence of 4.2% [12].

Urbanisation has been identified as the key driver of the evolving NCD epidemic in developing countries [13]. Identified lifestyle changes include reduced decreased physical activity; increase in smoking; increased alcohol intake. These are the behavioural risk factors for diabetes, which can lead to development of metabolic risk factors such as obesity. Since 2008, and for the first time in human history, the majority of the world’s population has lived in urban areas [14]. In Kenya while 22.3% of the population is urban, the urban population growth rate is 4.2% almost double the national population growth rate of 2.4% [15]. In Nairobi a majority (60%) of the population live in slums with much of the migrant population settling in slums. Further 75% of the urban population growth is absorbed by informal settlements. It is estimated that the number of urban population living in slums will double in the next 10 years [16]. Yet three quarters of the urban slum dwellers are deprived poor, living under impoverished conditions.

Despite this large and growing proportion of the population, there is a paucity of documentation on their burden of NCD. Our objective in this survey of the largest Nairobi slum was to determine the prevalence of diabetes mellitus and correlates such as physical activity, tobacco consumption; alcohol intake and metabolic risk factors such as hypertension and obesity.

## Method

The urban slum of Kibera in Nairobi, the capital of Kenya was chosen as the study area. Kibera is located 5 km southwest of Nairobi city centre and is approximately 2.5 square kilometres, with an estimated 300,000 inhabitants and density of 49,228 persons per square kilometre [15]. This study was conducted using UN recommendation for cluster sampling with probability proportional to size adjusted to achieve our sample size [17]. A design effect of 2.0 was used to account for the clustering of the study participants. A sampling frame with the list of villages and the projected populations of each was obtained. As a result a total of 80 clusters each containing 10 households and thus 25 participants per cluster were created within the eight villages with the number of clusters proportional to the population size within the particular village. The households in each cluster were visited in a random walk method from the nearest health centre, church or school as the focal point in each cluster. Within each household, all consenting adults 18 years and above who had been residents of the area for more than 3 months were enrolled. Pregnant women were excluded on account of the transient nature of gestational diabetes mellitus. The next household was taken as the one nearest to that previously visited until the sample size for the given cluster was achieved. The WHO STEPS instrument for collecting surveillance data for non-communicable diseases (NCD’s) was adopted to collect data.The WHO STEP wise approach to surveillance of non-communicable diseases is a validated instrument developed by WHO for collection of surveillance data on NCDs in resource poor settings. It is a sequential process made up of three main sections, which are the risk stratification questionnaire (step1), anthropometric measurements (step 2) and biochemical measurements (step3) [18,19]. The WHO steps instrument is attached as Additional file 1.

The survey was conducted between June and August 2010 with research teams visiting selected household between 8 am and 4 pm daily on weekdays. We minimized missing respondents by making a maximum of two revisits on subsequent Sundays. The questionnaire was administered by study trained registered medical assistants teams supervised by a study medical doctor. The questionnaire covered the smoking habits, alcohol use and physical activity pattern, history of prior evaluation for diabetes, and if any, the medication and lifestyle counselling that had been given. Anthropometric measures were recorded. Height was measured to the nearest 0.5 cm using a metal measuring tape against a wall and a flat headboard at right angles to the wall. Weight was determined using a good quality bathroom scale with the subject in light clothing and without shoes. Mid upper arm circumference was measured to the nearest 0.5 cm. Waist circumference was taken with a flexible tape measure placed on a horizontal plane at the level midpoint of the superior border of the iliac crest and the inferior margin of the last rib mid-axillary plane and the recording was at the point of normal expiration. Body mass index (BMI), calculated as weight (kg)/height (m^2^), was used as a measure of total body obesity while waist circumference and waist: hip ratio was used as measures of abdominal obesity. BMI <18.5 was recorded as underweight, 18.5-24.9 as normal, 25–29.9 as overweight and BMI more than 30 was recorded as obesity. Significant waist circumference was recorded as more than 102 cm (40 inches) for males and more than 88 cm (35 inches) in females. Significant waist to hip ratio was considered abnormal in females with a ratio of >0.8 and males >0.9.

Blood pressure was recorded using a mercury sphygmomanometer after the risk factor questionnaire had been filled to ensure that subjects had been seated for at least fifteen minutes. American Heart Association guidelines for measuring blood pressure were used. Three intermittent readings were taken and an average obtained. A random capillary blood sugar (RCBS) was recorded using a glucometer. Participants who had a RCBS >11.1 were invited to present themselves to the nearest health facility at a later designated date after a minimal 8 hours overnight fast. A fasting specimen of blood (five millilitres (5 mls) was drawn from a peripheral vein preferably the ante-cubital fossa and the serum was assayed for fasting venous blood sugar.

Research assistants underwent training on completion of the STEPS questionnaire and on anthropometric measurements to ensure standardization. The sphygmomanometers, weighing scales and tape measures were assessed weekly by taking measurements of one person on each of the instruments to ensure they were standardized. Recommended procedures for specimen collection, preparation and storage were followed to minimize pre-analytical errors. Before analysis, all the assays were calibrated according to the manufacturer’s specifications. Commercial controls used to validate the calibrations. Results were transcribed onto data sheets, which were checked by two people to minimize post analytical transcriptional errors.

Participant were diagnosed with diabetes if they had a RCBS level of >/= 11.1 mmol/l and a venous FBS of >/= 7.0 mmol/l, or had been diagnosed with diabetes or were receiving treatment for diabetes with insulin or oral hypoglycaemic agents. Hypertension was defined and classified as per the Seventh Report of the Joint National Committee [20] on prevention, detection and treatment of high blood pressure as being systolic BP ≥ 140 mmHg and/or diastolic BP ≥90 mmHg or use of prescribed antihypertensive medication. Hypertension was classified into pre-hypertension, stage 1 or 2 hypertension as per JNC VII. If the systolic and diastolic pressure readings belonged to different categories, the higher of the two readings was used to assign the blood-pressure stage.

### Statistical analysis

Statistical analysis was undertaken using Statistical Package for the Social Sciences (SPSS) version 16.0. Prevalence was age standardised utilising the new WHO world standard population [21]. Associations between the subjects socio-demographic, clinical and laboratory characteristics were examined using chi-square test for the categorical data while for continuous variables the Student *t*-test was used to determine statistical significance and Mann Whitney *U* test used in the analysis where such continuous data were skewed. Adjusted odds ratios for the association between diabetes mellitus and body mass indices and hypertension were computed, with 95% CIs. Associations were considered significant at p value less than or equal to 0.05.

### Ethical considerations

Study approval was obtained from the Kenyatta National Hospital (KNH) Ethics and Research Committee and the Ministry of Science and Technology. Administrative permission was obtained from the Nairobi City Council and the provincial administration and Kibera community leaders and elders were informed of the study. Follow up care for clinical conditions detected were facilitated by referral to nearby Mbagathi district Hospital or the national referral hospital as appropriate.

## Results

From a total of 936 households in eight villages, 2200 individuals were screened for eligibility, 2061 were enrolled and 139 excluded (38 pregnant, 50 declined consent and 51 below 18 years of age). We thus sampled on average 2.4 adults per household, however complete data was available in 2045 participants (99%). On account of insecurity incident during the survey one village (Laini Saba) was not visited.

Males comprised 50.9% and age ranged from 18 to 90 years with a mean age of 33.4 years (SD 11.6 years). Respondents were generally young with 53.9% aged between 25–44 years, 28.3% were under 24 years and only 5.2% were 55 years or older. Literacy level was high with 87% having either a primary or secondary level of educational attainment and the mean duration of years spent in school was 9.3 years.

Behavioural risk factors and demographic characteristics of the study sample are depicted in Table 1. Current cigarette smokers comprised 13.1% of whom 84.8% were daily smokers. The mean age of smoking commencement and duration of smoking was 19.7 years and 16.5 years respectively with a median of six-pack years. A larger proportion of males were smokers.

**Table 1:** Demographics and behavioural risk factors by sex

**Variable**	**Overall**	**Male**	**Female**
	% (**n**)	% (**n**)	% (**n**)
**Age yrs**			
18-24	28.3 (578)	27.7 (291)	28.8 (287)
25-34	31.8 (651)	30.1 (316)	33.7 (335)
35-44	22.1 (452)	22.2 (233)	22.0 (219)
45-54	12.5 (256)	13.7 (144)	11.3 (112)
55-64	3.8 (78)	4.8 (50)	2.8 (28)
65-74	1.2 (25)	1.4 (15)	1.0 (10)
≥ 75	0.2 (5)	0.1 (1)	0.4 (4)
18-90	100 (2045)	51.0 (1050)	48.4 (995)
**Years in school**, **mean (SD)**	9.3 (3.3)	9.8 (3.2)	8.7 (3.2)
**Tobacco smoking**			
Current % (n)	13.1 (269)	22 (231)	3.8 (38)
Smoking daily % (n)	84.8 (228)	89.2 (206)	57.9 (22)
Age started yrs mean (SD)	19.7 (5.5)	19.9 (5.7)	18.6 (3.4)
Age started range yrs	10-45	10-45	10-24
Duration years mean (SD)	16.5 (10)	16.4 (10.2)	17.3 (9.1)
Pack years median (IQR)	6 (2.5-10.9)	6 (2.4-10.5)	7.5 (4.4-12.0)
**Alcohol consumption**			
Ever consumed	30.1 (616)	43.2 (454)	16.3 (162)
In past 12 months	81.0 (499)	89.6 (407)	56.8 (92)
In past 30 days	76.8 (383)	79.1 (322)	66.3 (61)
Frequency in past 12 months			
Daily	19.1 (95)	22.1 (90)	5.5 (5)
5-6 days/week	14.3 (71)	13.8 (56)	16.5 (15)
1-4 days/week	28.7 (143)	27.0 (110)	36.3 (33)
1-3 days/week	23.3 (116)	22.9 (93)	25.3 (23)
Average number of drinks median	4 (3–6)	4 (3–6)	3 (2–5)
(IQR)	6 (4.5-9)	6 (5–9)	6 (4–7)
Largest number of drinks/sitting			
**Physical Activity**			
**Work Vigorous**	29.6 (606)	39.1 (411)	19.6 (195)
Days/week median(IQR)	6 (5–7)	6 (5–7)	6 (3–7)
Hours/day median (IQR)	8 (3–9)	8 (4–9)	6 (2–8)
**Work moderate**	46.1 (943)	39.1 (411)	53.4 (532)
Days/week median(IQR)	6 (5–7)	6 (4–7)	7 (5–7)
Hours/day median (IQR)	6 (3–8)	6 (3–8)	5 (2.1-8.0)
**Travel** (**walk**/**cycle**)	77.4(1583)	80.3 (843)	74.3 (740)
Days/week median(IQR)	6 (5–7)	6 (5–7)	6 (4–7)
Hours/day median (IQR)	1 (0.75-2)	1.3 (0.8-2.0)	1 (0.7-2.0)
**Recreational vigorous**	15.2 (311)	21.7 (228)	8.3 (83)
Days/week median(IQR)	2.0 (1–3)	2.0 (1.0-3.0)	2 (1–3)
Hours/day median (IQR)	2.0 (0.8-2)	2.0 (1.0-3.0)	1.4 (1–2)
**Recreational moderate**	16.4 (336)	2.0 (1.0-2.0)	16 (159)
Days/week median(IQR)	3 (1–6)	16.9 (177)	3 (1–6)
Hours/day median (IQR)	1 (0.7-2)	3.0 (1.0-6.0)	1 (0.7-2)
Time sitting/reclining hrs mean(range) ^+^	4 (2–6)	1.5 0.8-2.0)	4 (2–7)
METS*, median (IQR)	10800 (3840–21120)	4.0(2–6) 13474 (5040–24960)	8400 (3168–15630)

^+^ sleeping time not included.

* METs minutes per week.

The study demonstrated a high level of alcohol consumption with 30% reported to have ever consumed alcohol; 74.9% of whom consumed alcohol in the previous twelve months and 62.2% in the previous 30 days of study. The frequency of consumption in the preceding twelve months was 19.7% on a daily basis and 43.4% between 1–6 days per week; with the average and largest number of drinks per sitting being four and six drinks respectively. Males demonstrated a higher consumption level of alcohol than their female counter parts.

As anticipated study subjects demonstrated a high level of physical activity (PA) that was predominantly work and travel related; with 75.7% (male 79.1%, female 73%) undertaking vigorous or moderate work related activity, for a median duration of six hours per day; and 77.4% (male 80.3%, female 74.3%) walking or cycling as mode of transport; the latter at a median frequency of 6 days per week. This is also demonstrated by METS minutes per week data. Vigorous work related PA was more common in males and the converse for moderate work related PA (Table 1).

Anthropometric measures of body weight status, depicted in Table 2, was available for 98% of participants (Body Mass Index (BMI) n = 2036 Waist circumference (WC) n = 2031 and Waist Hip Ratio (WHR) n = 2026 Majority had a normal body weight measure of BMI, WC and WHR (50.1%, 78.5% and 86.7% respectively), however overall 16.3% were obese and 29% overweight by BMI and 21.5% and 13.3% had an elevated WC and WHR. An elevated body mass by all measures was more frequent in women, with that for obesity, WC and WHR reaching statistical significance. Remarkably the proportion of women with obesity was 32.2%, central obesity 41.5% and elevated WHR 24%. Overweight status did not show a sex differential.

**Table 2:** Distribution of body mass by sex

**Variable**	**ALL % (n)**	**Male % (n)**	**Female% (n)**	**P value**
**BMI Kg**/**m**^**2**^				
< 18.5	4.5 (92)	5.8 (61)	3.1 (31)	0.476
18.5 – 25	50.1 (1020)	61.0 (638)	38.5 (382)	Ref
>25 -29.9	29.1 (592)	26.1 (273)	32.2 (319)	<0.001
≥ 30	16.3 (333)	7.1 (74)	26.1 (259)	<0.001
**Waist Circ**. (**cm**)				
Elevated	21.5 (437)	2.6 (27)	41.5 (410)	<0.001
Normal	78.5 (1595)	97.4 (1017)	58.5 (578)	
**Waist Hip Ratio**				
High	13.3 (269)	3.2 (33)	24.0 (236)	<0.001
Normal	86.7 (1758)	96.8 (1009)	76.0 (749)	

Waist Circumference (Waist Circ): Elevated Male >102 cm; Female > 88 cm.

Waist Hip Ratio High: >0.90 males and 0.80 in females.

A diagnosis of Diabetes was established in 3.2% (n 66; 95% CI 2.5-4.0) of screened subjects; 53% (n 35) screen detected and 47% (n 31) previously diagnosed. A meager 10.6% of those without-diabetes had ever in their lifetime had a blood sugar measurement. The age adjusted prevalence of diabetes was 5.3% (95% CI 4.2-6.4). Prevalence increased with age peaking at 10.5% (n 27; 95% CI 6.8-14.3%) in the 45–54 yr. age category, though the rates in those under 24 years and in the more advanced age categories were unstable on account of small numbers (Table 3). The female male ratio was 1.3: 1.0, however prevalence was not statistically different among sex in categories with stable rates. Therapy in use among those with diabetes was 58.8% dietary, 48.4% oral hypoglycaemic agents, 22.6% insulin and 12.9% herbal therapy.

**Table 3:** Prevalence of diabetes mellitus across age and sex

**Variable**	**Prevalence (95% CI)**	**Male (95%CI)**	**Female (95% CI)**	**P value**
**Age yrs**				
18-24	0.3 (−0.1-0.8)	0.7 (−0.3-1.6)	0.0	0.499
25-34	1.5 (0.6-2.5)	1.2 (0.0-2.5)	1.8 (0.4-3.2)	0.753
35-44	3.3 (1.7-5.0)	2.6 (1.0-5.5)	4.1 (2.0-7.7)	0.363
45-54	10.5 (6.8-14.3)	8.2 (3.7-12.7)	13.4 (7.0-19.7)	0.191
55-64	7.7 (1.7-13.6)	2.0 (0.1-10.7)	17.9 (6.1-36.9)	0.021
≥ 65	20.0 (5.4-34.6)	25.0 (3.1-46.9)	14.3 (0.0-33.3)	0.657
18-90	3.2 (2.5-4.0)	2.8 (1.9-4.0)	3.7 (2.7-5.1)	0.223

Table 4 depicts the distribution of behavioural risk factors by diabetic and sex status. The proportion of persons with diabetes with the behavioural risk factors was high and mirrored that of the entire sample; at cigarette smoking 12.1% and alcohol consumption 21.2%, however persons with diabetes had a significantly 12 year longer duration of cigarette smoking (28.6 years; 16 years; p 0.0001) and double the pack years of persons without diabetes (12.8;6.0; p 0.049). Persons with diabetes demonstrated similarly high physical activity levels to persons without diabetes.

**Table 4:** Risk factor distribution by diabetic status

**Variable**	**All % (n)**	**Persons with diabetes**	**Non without diabetes**	**P value**
**Tobacco Smoking**				
Current %(n)	13.2 (269)	12.1 (8)	13.2 (261)	0. 801
Smoking daily %(n)	84.8 (228)	87.5 (7)	84.7 (221)	1.000
Age Started yrs mean(SD)	19.7 (5.5)	20.1 (1.5)	19.7 (5.6)	0.844
Duration Smoking yrs mean (SD)	16.5 (10)	28.6 (11.6)	16.0 (10)	0.001
Pack years median(IQR)	6.0 (2.5-10.9)	12.8 (8.8-20.3)	6 (2.5-10.5)	0.049
**Alcohol Consumption**				
Ever consumed	30.1 (616)	21.2 (14)	30.4 (602)	0.109
In past 12 months	81.0 (499)	92.9 (13)	80.7 (486)	0.487
In past 30 days	76.8 (383)	69.2 (9)	77 (374)	0.511
Frequency in past 12 months				
Daily	19.1 (95)	30.8 (4)	18.8 (91)	0.768
5-6 days/wk	14.3 (71)	7.7 (1)	14.4 (70)	
1-4 days/wk	28.7 (143)	30.8 (4)	28.7 (139)	
1-3 days/wk	23.3 (116)	23.1 (3)	23.3 (113)	
Avg. No. drinks median(IQR)	4 (3–6)	4 (4–6)	4 (3–6)	0.408
Largest No. drinks/sitting	6 (5–9)	8 (6–9)	6 (5–9)	0.367
**Physical Activity**				
**Work Vigorous**	29.6 (606)	19.7 (13)	30.0 (593)	0.072
Days/week median(IQR)	6 (5–7)	6 (3–7)	6 (5–7)	0.928
Hours/day median (IQR)	8 (3–9)	8 (4–10)	8 (3–9)	0.494
**Work moderate**	46.1 (942)	39.4 (26)	46.3 (916)	0.269
Days/week median(IQR)	6 (5–7)	4.5 (2–7)	6 (5–7)	0.005
Hours/day median (IQR)	6 (3–8)	5 (2–8)	6 (3–8)	0.136
**Travel** (**walk**/**cycle**)	77.4 (1582)	71.2 (47)	77.6 (1535)	0.225
Days/week median(IQR)	6 (5–7)	6(4–7)	6 (5–7)	0.741
Hours/day median (IQR)	1 (0.8-2)	1 (0.7-3.0)	1 (0.8-2)	0.867
**Recreational vigorous**	15.2 (311)	12.1 (8)	15.3 (303)	0.478
Days/week median(IQR)	2.0 (1–3)	4.5 (2–6)	2 (1–3)	0.084
Hours/day median (IQR)	2.0 (1–2)	2.5 (0.4-5)	2 (1–2)	0.418
**Recreational moderate**	16.4 (336)	21.2 (14)	16.3 (322)	0.287
Days/week median(IQR)	3 (1–6)	5 (3–7)	2 (1–6)	0.021
Hours/day median (IQR)	1 (0.8-2)	1 (0.6-3)	1 (0.8-2)	0.810
Time sitting/recining ^+^ hrs(range)	4 (2–6)	4.2 (2–8)	4 (2–6)	0.306
METS median (IQR)	10800 (3840–21120)	5760 (2011–20160)	10800 (3912–21120)	0.085

^+^ Sleeping time not included.

* METs minutes per week.

Body mass distribution by diabetes status and sex is depicted in Table 5. Compared to persons without, persons with diabetes were three fold (OR 3.2; 95% CI 1.7-6.2%) more likely to be obese, with no significant sex differences. Persons with diabetes were twice as likely to have an elevated WC (OR 2.3; 95% CI 1.2-4.6%) and WHR (OR 2.1; 95% 1.1-3.9%); with no significant sex differences detectable. This relationship between body mass and diabetes status was unchanged on adjusting for smoking, alcohol use and physical activity (MET). However the positive relationship between waist circumference and diabetes status was no longer significant on adjusting for smoking, alcohol use and physical activity (MET).

**Table 5:** Distribution of body mass indices by diabetes status

**Variable**	**Diabetes % (n)**	**Non diabetes % (n)**	**Odds ratio (95% CI)**	**P value**	**Age**-**adjusted OR (95% CI)**	**P value**	**Age-sex adjusted OR (95% CI)**	**P value**	**Age-sex-smoking**-**alcohol use-METS adjusted OR (95% CI)**	**P value**
**BMI** (**n 2036**)										
> 30 Obese										
Female	79.3 (23)	77.6 (235)	1.1 (0.4-2.8)	0.828	1.5 (0.5-4.0)	0.457				
Male	20.7 (6)	22.4 (68)	ref							
All	44.6 (29)	15.4 (303)	5.0 (2.8-9.1)	0.000	3.4 (1.8-6.3)	0.000	3.2 (1.7-6.2)	0.001	3.4 (1.5-7.5)	0.003
>25-29.9 Overweight										
Female										
Male	50 (7)	54 (312)	0.9 (0.3-2.5)	0.768	1.0 (0.3-3.0)	0.965				
All	50 (7)	46 (266)	Ref							
	21.5(14)	29.3 (578)	1.3 (0.6-2.6)	0.494	0.9 (0.5-1.9)	0.881	0.9 (0.4-1.9)	0.833	1.2 (0.6-2.7)	0.612
18.5-25 Normal										
Female	31.6 (6)	37.6 (376)	0.8 (0.3-2.0)	0.593	1.0 (0.4-2.6)	0.949				
Male	68.4 (13)	62.4 (625)	Ref							
All	29.2 (19)	50.8 (1001)	Ref							
< 18.5 Under weight										
Female	33.3 (1)	33.7 (30)	1.0 (0.1-11.3)	1.000	1.7 (0.1-22.9)	0.705				
Male	66.7 (2)	66.3 (59)	Ref							
All	4.6 (3)	4.5 (89)	1.8 (0.5-6.1)	0.363	1.9 (0.5-6.8)	0.308	1.9 (0.6-6.8)	0.301	3.0 (0.8-11.2)	0.101
**Waist Cir**. (**n 2031**)										
Elevated										
Female	89.7 (26)	94.1 (383)	0.5 (0.2-2.0)	0.441	1.0 (0.2-1.1)	0.977				
Male	10.3 (3)	5.9 (24)	Ref							
All	44.6 (29)	20.7 (407)	3.1 (1.9-5.1)	0.000	2.2 (1.3-3.7)	0.003	2.3 (1.2-4.6)	0.015	2.1 (1.0-4.5)	0.068
Normal										
Female	27.8 (10)	36.4 (568)	0.7 (0.3-1.4)	0.285	0.9 (0.4-2.0)	0.828				
Male	72.2 (26)	63.6 (991)	Ref							
All	55.4 (36)	79.2 (1559)	Ref							
**Waist Hip Ratio** (**n 2026**)										
High										
Female	85 (17)	88 (219)	0.8 (0.2-2.8)	0.721	0.9 (0.2-3.5)	0.897				
Male	15 (3)	12 (30)	Ref							
All	30.8 (20)	12.7 (249)	3.1 (1.8-5.3)	0.000	2.3 (1.3-4.0)	0.004	2.1 (1.1-3.9)	0.020	2.4 (1.1-5.0)	0.028
Normal										
Female	42.2 (19)	42.6 (729)	1.0 (0.5-1.8)	0.962	1.2 (0.7-2.3)	0.505				
Male	57.8 (26)	57.4 (983)	Ref							
All	69.2 (45)	87.3 (1712)								

Waist Circumference (Waist Circ): Elevated Male >102 cm; Female > 88 cm.

Waist Hip Ratio High: >0.90 males and 0.80 in females.

Overall 45.5% (n 30) of persons with diabetes were hypertensive; 43.9% (n 29) pre-hypertensive and only 10.6% (n 7) were normotensive; 53% (n 17/30) of hypertensives were classified as Stage I. The age, sex, smoking, alcohol and physical activity adjusted odds of a diabetic being hypertensive against being normotensive was 3.2 (OR 95% CI 1.6-6.1; 3.4 (OR 95% CI 1.1-10.0.) for Stage I hypertension and 6.0 (OR 95%CI 2.1-17.5) for Stage II hypertension (Table 6).

**Table 6:** Blood pressure distributions by sex and diabetes status

**Variable**	**Diabetes % (n)**	**Non diabetes % (n)**	**Odds ratio**	**P value**	**Age-adjusted OR (95% CI)**	**P value**	**Age-sex adjusted OR (95% CI)**	**P value**	**Age-sex**-**smoking-alcohol use-METS adjusted OR (95% CI)**	**P value**
**BP Classification**										
**Stage 2 Hypertension**										
Female	61.5 (8)	49.4 (39)	1.6(0.5-5.5)	0.416	1.6 (0.5-5.5)	0.426				
Male	38.5 (5)	50.6 (40)	Ref							
All	19.7 (13)	4.0 (79)	14.1 (5.5-36.5)	0.000	5.7 (2.1-15.4)	0.001	5.6 (2.1-15.3)	0.001	6.0 (2.1-17.5)	0.001
**Stage 1 Hypertension**										
Female	64.7 (11)	49.6 (61)	1.9 (0.7-5.4)	0.243	1.5 (0.5-4.6)	0.443				
Male	35.3 (6)	50.4 (62)	Ref							
All	25.8 (17)	6.2 (123)	12.0 (4.9-29.6)	0.000	6.3 (2.5-16.1)	0.000	6.2 (2.4-15.8)	0.001	3.4 (1.1-10.0)	0.029
**Pre**- **Hypertension**										
Female	48.3 (14)	46.2 (540)	1.1 (0.5-2.3)	0.824	1.3 (0.6-2.7)	0.520				
Male	51.7 (15)	53.8 (629)	Ref							
All	43.9 (29)	59.2 (1169)	2.1 (0.9-4.9)	0.073	1.8 (0.8-4.1)	0.181	1.8 (0.8-4.2)	0.167	1.6 (0.7-3.8)	0.290
**Hypertension**										
Female	63.3 (19)	49.5 (100)	1.8 (0.8-3.9)	0.157	1.6 (0.7-3.6)	0.256				
Male	36.7 (11)	50.5 (102)	Ref							
All	45.5 (30)	10.2 (202)	7.3 (4.4-12.1)	0.000	3.9 (2.2-6.7)	0.000	3.8 (2.2-6.5)	0.000	3.2 (1.6-6.1)	0.001
**Normal**										
Female	50.0 (18)	48.3 (856)	1.1 (0.6-2.1)	0.841	1.3 (0.7-2.5)	0.459				
Male	50.0 (18)	51.7 (916)	Ref							
All	54.5 (36)	89.3 (1772)	Ref							

## Discussion

We set out in this first Kenyan urban-poor population survey to determine the prevalence of diabetes and associated behavioural and metabolic risk factors in the largest growing segment of our urban population, a population hitherto deemed to be burdened with infectious diseases and under nutrition. In this initial report we demonstrate a high age adjusted prevalence of predominantly survey detected, diabetes mellitus. The prevalence of diabetes increased with age and showed no sex differential. This is occurring in an unplanned high-density urban settlement community; of relative young age structure, with high levels of self-reported daily life related physical activity. However, these slum residents are exposed to high level of behavioural risk factors such as cigarette smoking and harmful alcohol consumption, the latter occurring in over two thirds of subjects. Close to half were either obese or overweight; a fifth had an elevated waistline and high body mass indices both of which were more frequent in females.

A higher prevalence of type 2 diabetes mellitus in deprived communities has been documented and is most striking in middle age (40–49 yr.) [6,22,23], which mirrors the highest age category specific prevalence in our data. The occurrence of DM in persons of low socio-economic status has been attributed to dietary patterns, low physical activity, cigarette smoking and low birth weight [24]. Our data documents a high level of physical activity (PA) activity; moderate levels of obesity and overweight; moderate smoking; and heavy alcohol use thus suggesting that dietary factors are a significant contributor.

Screen detection rate of diabetes mellitus in communities has been shown to be a marker of availability of health care facilities [25], and is suggested in our sample by only 10% having ever had a blood sugar measured. High screen detection rates of 70-100% have been reported in SSA and 50% from urban South Africa [10,26]. We report a similarly high rate, with 50% of our sample being undetected, in a population of relatively high literacy compared to the national adult literacy of 61.5% [27] implying that contributing factors are probably low access to primary health care facilities and low public awareness of diabetes mellitus. This raises the need for evaluation of these factors alongside preventive strategies of screening high-risk groups. Our data point to the high-risk groups of hypertension, that was significantly associated with the presence of DM, and general and central obesity. Family history and impaired glucose tolerance are other high-risk markers.

Behavioural risk factors among the diabetic subjects were similar to that of the entire sample, however persons with diabetes were three fold more likely to be have generalised obesity and hypertension. Central obesity as measured by waist circumference was not predictive of diabetes on adjusting for covariates of physical activity. This data suggests that our study population’s transition to higher cardio-metabolic risk is presently being driven predominantly by dietary factors and less so by physical inactivity and other behavioural determinants. Despite the absence of longitudinal data on the prevalence of diabetes in Kenya our documented high DM disease burden rate in slum dwellers is suggestive of a rapidly occurring reversal of the social class gradient of CVD risk factors in urban Kenya [24].

A further important potential consequence of a high prevalence of diabetes among the urban high density poor is the association with communicable diseases, particularly tuberculosis and therefore HIV [26]. In other parts of the developing world diabetes has been shown to be associated with a threefold increase risk of tuberculosis [28] and one in five cases of new smear positive tuberculosis in India has been attributed to diabetes [29]. The recognition of diabetes link with poverty is thus vital as it has important implications in the prevention and care in these settings.

The all age worldwide prevalence of diabetes mellitus in 2000 was estimated at 2.8% and is projected to rise to 4.4% by 2030 [30]. In sub-Saharan Africa (SSA) type 2 diabetes mellitus is the most frequent type and although data is scarce prevalence has been shown to be rising over the last two decades with highest frequencies in urban areas. No comparative slum/urban poor data is available from Kenya. However utilising the 1998 WHO criteria, Mathenge in a 2010 survey of adults over the age of 50 years in an urban and rural parts of a cosmopolitan district in Kenya report a non-age adjusted prevalence of 6.6% [31]. The only other contemporary data from Kenya is that of a mixed location opportunity sample in person over 18 years, using OGTT that reported an age standardised rate of 4.2% [12]. Studies from other part of SSA report similar urban age adjusted rates of between 5.9-6.4% [10]. These rates are consistent with our findings, which we believe to be an underestimate in the absence of oral glucose tolerance testing. The economic and social consequences of diabetes are expected to be greatest among the poor; reportedly utilising up to 25% of their income on care and predominantly affecting the breadwinners of these communities [32].

### Strengths and limitations

To the best of our knowledge this is the first published urban slum NCD survey report from Kenya, with a stable age adjusted standardised prevalence rates, in a representative sample and utilising standard methods that allow for comparability of data across studies and regions. Relying predominantly on a fasting capillary blood sugar and exclusion of pregnant women may have contributed to a prevalence underestimate. Over representation of large families has potential to overestimate the prevalence, however we sampled 2.4 adults per household in an area with an average household size of 3.2 and thus we do not believe that family size significantly affects our results [33]. We do not have evidence that the exclusion of one village due to insecurity would have had an effect on the estimate of prevalence.

The availability of dietary and socio-economic data would have strengthened our findings. Our physical activity data suffers from the limitation of being self-reported, even though the STEPS questionnaire has been validated in similar populations, and may represent a bi-directional measurement bias.

## Conclusions

In conclusion we have demonstrated that this, previously unstudied urban slum community has a high prevalence of diabetes mellitus and associated risk factors signalling a lack of access to care and low public awareness in a highly literate but poor population. This sign of incomplete epidemiological transition has implications for transmission of infectious disease such as TB and consequences of increasing healthcare costs. These are the people suffering from the double burden of disease [34].

It is our recommendation that for this rapidly expanding segment of our population policy makers need to focus their attention on strategies that address not just communicable diseases but non-communicable diseases as well.

## Competing interests

The authors declare that they have no competing interests.

## Authors’ contributions

RA conceived of the study, participated in the design, performed the statistical analysis and drafted the manuscript. MJ conceived the study, participated in the design of the study, performed the statistical analysis and drafted the manuscript. RW conceived of the study, participated in the design performed the statistical analysis and helped draft the manuscript. EKN participated in the design of the study and performed the statistical analysis. CFO participated in the design and helped draft the manuscript. KKM performed the statistical analysis and helped draft the manuscript. All authors read and approved the final manuscript.

## Pre-publication history

The pre-publication history for this paper can be accessed here:

http://www.biomedcentral.com/1471-2458/13/371/prepub

## Supplementary Material

Additional file 1 WHO STEPS Instrument (Core and Expanded). Click here for file
