# Supplementary material for: A population-based survey of prevalence of diabetes and correlates in an urban slum community in Nairobi, Kenya
Source: BMC Public Health. 2013 Apr 20;13:371. doi: 10.1186/1471-2458-13-371 (PMC3641964; doi:10.1186/1471-2458-13-371)
Supplement: Additional file 1 — WHO STEPS Instrument (Core and Expanded). [file 1471-2458-13-371-S1.pdf]

WHO STEPS Instrument

(Core and Expanded)

The WHO STEPwise approach to chronic disease risk factor surveillance (STEPS)

World Health Organization
20 Avenue Appia, 1211 Geneva 27, Switzerland

For further information: www.who.int/chp/steps

World Health Organization

WHO STEPS Instrument
for Chronic Disease Risk Factor Surveillance

<insert country/site name>

Survey Information

Location and Date		Response	Code
1	Cluster/Centre/Village ID		I1
2	Cluster/Centre/Village name		I2
3	Interviewer ID		I3
4	Date of completion of the instrument	dd mm year	I4

Participant Id Number

Consent, Interview Language and Name		Response	Code
5	Consent has been read and obtained	Yes 1<br>No 2 **If NO, END**	I5
6	Interview Language *[Insert Language]*	English 1<br>*[Add others]* 2<br>*[Add others]* 3<br>*[Add others]* 4	I6
7	Time of interview<br>(24 hour clock)	: hrs mins	I7
8	Family Surname		I8
9	First Name		I9
**Additional Information that may be helpful**			
10	Contact phone number where possible		I10

Record and file identification information (I5 to I10) separately from the completed questionnaire.

Step 1 Demographic Information

CORE: Demographic Information

Question		Response	Code
11	Sex (*Record Male / Female as observed*)	Male 1<br>Female 2	C1
12	What is your date of birth?<br>*Don't Know 77 77 7777*	*If known, Go to C4*<br>dd mm year	C2
13	How old are you?	Years	C3
14	In total, how many years have you spent at school or in full-time study (excluding pre-school)?	Years	C4

Step 1 Behavioural Measurements

CORE: Tobacco Use

Now I am going to ask you some questions about various health behaviours. This includes things like smoking, drinking alcohol, eating fruits and vegetables and physical activity. Let's start with tobacco.

Question		Response	Code
22	Do you currently smoke any **tobacco products**, such as cigarettes, cigars or pipes? *(USE SHOWCARD)*	Yes 1<br>No 2 *If No, go to T6*	T1
23	Do you currently smoke tobacco products **daily**?	Yes 1<br>No 2 *If No, go to T6*	T2
24	How old were you when you **first started** smoking daily?	Age (years) └─┴─┘ *If Known, go to T5a*<br>Don't know 77	T3
25	Do you remember how long ago it was?<br>*(RECORD ONLY 1, NOT ALL 3)*<br>*Don't know 77*	In Years └─┴─┘ *If Known, go to T5a*	T4a
		OR in Months └─┴─┘ *If Known, go to T5a*	T4b
		OR in Weeks └─┴─┘	T4c
26	On average, **how many** of the following do you smoke each day?<br>*(RECORD FOR EACH TYPE, USE SHOWCARD)*<br>*Don't Know 77*	Manufactured cigarettes └─┴─┘	T5a
		Hand-rolled cigarettes └─┴─┘	T5b
		Pipes full of tobacco └─┴─┘	T5c
		Cigars, cheroots, cigarillos └─┴─┘	T5d
		Other └─┴─┘ *If Other, go to T5other, else go to T9*	T5e
		Other (please specify): ________ *Go to T9*	T5other

CORE: Alcohol Consumption

The next questions ask about the consumption of alcohol.

Question		Response	Code
36	Have you **ever** consumed an alcoholic drink such as beer, wine, spirits, fermented cider or *[add other local examples]*?<br>*(USE SHOWCARD OR SHOW EXAMPLES)*	Yes 1<br>No 2 *If No, go to D1*	A1a
37	Have you consumed an alcoholic drink within the **past 12 months**?	Yes 1<br>No 2 *If No, go to D1*	A1b
38	During the past 12 months, **how frequently** have you had at least one alcoholic drink?<br>*(READ RESPONSES, USE SHOWCARD)*	Daily 1<br>5-6 days per week 2<br>1-4 days per week 3<br>1-3 days per month 4<br>Less than once a month 5	A2
39	Have you consumed an alcoholic drink within the **past 30 days**?	Yes 1<br>No 2 *If No, go to D1*	A3
40	During the past 30 days, on how many **occasions** did you have at least one alcoholic drink?	Number<br>Don't know 77	A4
41	During the past 30 days, when you drank alcohol, **on average**, how many **standard alcoholic drinks** did you have during one drinking occasion?<br>*(USE SHOWCARD)*	Number<br>Don't know 77	A5

42	During the past 30 days, what was the **largest number** of standard alcoholic drinks you had on a single occasion, counting all types of alcoholic drinks together?	Largest number<br>Don't Know 77	A6
43	During the past 30 days, how many times did you have for **men**: **five or more** for **women**: **four or more** standard alcoholic drinks in a single drinking occasion?	Number of times<br>Don't Know 77	A7

CORE: Physical Activity

Next I am going to ask you about the time you spend doing different types of physical activity in a typical week. Please answer these questions even if you do not consider yourself to be a physically active person.
Think first about the time you spend doing work. Think of work as the things that you have to do such as paid or unpaid work, study/training, household chores, harvesting food/crops, fishing or hunting for food, seeking employment. *[Insert other examples if needed].* In answering the following questions 'vigorous-intensity activities' are activities that require hard physical effort and cause large increases in breathing or heart rate, 'moderate-intensity activities' are activities that require moderate physical effort and cause small increases in breathing or heart rate.

Question		Response	Code
**Work**			
52	Does your work involve vigorous-intensity activity that causes large increases in breathing or heart rate like *[carrying or lifting heavy loads, digging or construction work]* for at least 10 minutes continuously?<br>*[INSERT EXAMPLES] (USE SHOWCARD)*	Yes 1<br>No 2 *If No, go to P 4*	P1
53	In a typical week, on how many days do you do vigorous-intensity activities as part of your work?	Number of days	P2
54	How much time do you spend doing vigorous-intensity activities at work on a typical day?	Hours : minutes : hrs mins	P3 (a-b)
55	Does your work involve moderate-intensity activity, that causes small increases in breathing or heart rate such as brisk walking *[or carrying light loads]* for at least 10 minutes continuously?<br>*[INSERT EXAMPLES] (USE SHOWCARD)*	Yes 1<br>No 2 *If No, go to P 7*	P4
56	In a typical week, on how many days do you do moderate-intensity activities as part of your work?	Number of days	P5
57	How much time do you spend doing moderate-intensity activities at work on a typical day?	Hours : minutes : hrs mins	P6 (a-b)
**Travel to and from places**			
The next questions exclude the physical activities at work that you have already mentioned.<br>Now I would like to ask you about the usual way you travel to and from places. For example to work, for shopping, to market, to place of worship. *[Insert other examples if needed]*			
58	Do you walk or use a bicycle *(pedal cycle)* for at least 10 minutes continuously to get to and from places?	Yes 1<br>No 2 *If No, go to P 10*	P7
59	In a typical week, on how many days do you walk or bicycle for at least 10 minutes continuously to get to and from places?	Number of days	P8
60	How much time do you spend walking or bicycling for travel on a typical day?	Hours : minutes : hrs mins	P9 (a-b)

CORE: Physical Activity, Continued

	Question	Response	Code
**Recreational activities**			
The next questions exclude the work and transport activities that you have already mentioned.<br>Now I would like to ask you about sports, fitness and recreational activities (leisure), *[Insert relevant terms]*.			
61	Do you do any vigorous-intensity sports, fitness or recreational *(leisure)* activities that cause large increases in breathing or heart rate like *[running or football]* for at least 10 minutes continuously?<br>*[INSERT EXAMPLES] (USE SHOWCARD)*	Yes 1<br>No 2 *If No, go to P 13*	P10
62	In a typical week, on how many days do you do vigorous-intensity sports, fitness or recreational *(leisure)* activities?	Number of days □□	P11
63	How much time do you spend doing vigorous-intensity sports, fitness or recreational activities on a typical day?	Hours : minutes □□ : □□<br>hrs mins	P12 (a-b)
64	Do you do any moderate-intensity sports, fitness or recreational *(leisure)* activities that cause a small increase in breathing or heart rate such as brisk walking, *[cycling, swimming, volleyball]* for at least 10 minutes continuously?<br>*[INSERT EXAMPLES] (USE SHOWCARD)*	Yes 1<br>No 2 *If No, go to P16*	P13
65	In a typical week, on how many days do you do moderate-intensity sports, fitness or recreational *(leisure)* activities?	Number of days □□	P14
66	How much time do you spend doing moderate-intensity sports, fitness or recreational *(leisure)* activities on a typical day?	Hours : minutes □□ : □□<br>hrs mins	P15 (a-b)

EXPANDED: Physical Activity

**Sedentary behaviour**			
The following question is about sitting or reclining at work, at home, getting to and from places, or with friends including time spent sitting at a desk, sitting with friends, traveling in car, bus, train, reading, playing cards or watching television, but do not include time spent sleeping.<br>*[INSERT EXAMPLES] (USE SHOWCARD)*			
67	How much time do you usually spend sitting or reclining on a typical day?	Hours : minutes □□ : □□<br>hrs mins	P16 (a-b)

CORE: History of Raised Blood Pressure

	Question	Response	Code
68	Have you ever had your blood pressure measured by a doctor or other health worker?	Yes 1<br>No 2 *If No, go to H6*	H1
69	Have you ever been told by a doctor or other health worker that you have raised blood pressure or hypertension?	Yes 1<br>No 2 *If No, go to H6*	H2a
70	Have you been told in the past 12 months?	Yes 1<br>No 2	H2b

**EXPANDED: History of Raised Blood Pressure**			
71	Are you currently receiving any of the following treatments/advice for high blood pressure prescribed by a doctor or other health worker?		
	Drugs (medication) that you have taken in the past two weeks	Yes 1<br>No 2	H3a
	Advice to reduce salt intake	Yes 1<br>No 2	H3b
	Advice or treatment to lose weight	Yes 1<br>No 2	H3c
	Advice or treatment to stop smoking	Yes 1<br>No 2	H3d
	Advice to start or do more exercise	Yes 1<br>No 2	H3e
72	Have you ever seen a traditional healer for raised blood pressure or hypertension?	Yes 1<br>No 2	H4
73	Are you currently taking any herbal or traditional remedy for your raised blood pressure?	Yes 1<br>No 2	H5

**CORE: History of Diabetes**			
**Question**		**Response**	**Code**
74	Have you ever had your blood sugar measured by a doctor or other health worker?	Yes 1<br>No 2 *If No, go to M1*	H6
75	Have you ever been told by a doctor or other health worker that you have raised blood sugar or diabetes?	Yes 1<br>No 2 *If No, go to M1*	H7a
76	Have you been told in the past 12 months?	Yes 1<br>No 2	H7b

**EXPANDED: History of Diabetes**			
77	Are you currently receiving any of the following treatments/advice for diabetes prescribed by a doctor or other health worker?		
	Insulin	Yes 1<br>No 2	H8a
	Drugs (medication) that you have taken in the past two weeks	Yes 1<br>No 2	H8b
	Special prescribed diet	Yes 1<br>No 2	H8c
	Advice or treatment to lose weight	Yes 1<br>No 2	H8d
	Advice or treatment to stop smoking	Yes 1<br>No 2	H8e
	Advice to start or do more exercise	Yes 1<br>No 2	H8f
78	Have you ever seen a traditional healer for diabetes or raised blood sugar?	Yes 1<br>No 2	H9
79	Are you currently taking any herbal or traditional remedy for your diabetes?	Yes 1<br>No 2	H10

Step 2 Physical Measurements

Question		Response		Code
**CORE: Height and Weight**				
80	Interviewer ID		□□□□□□□	M1
81	Device IDs for height and weight	Height	□□□□□	M2a
		Weight	□□□□□	M2b
82	Height	in Centimetres (cm)	□□□□□□□. □□□	M3
83	Weight<br>*If too large for scale 666.6*	in Kilograms (kg)	□□□□□□□.□□□	M4
84	**For women:** Are you pregnant?	Yes	1 *If Yes, go to M 8*	M5
		No	2	
**CORE: Waist**				
85	Device ID for waist		□□□□□	M6
86	Waist circumference	in Centimetres (cm)	□□□□□□□.□□□	M7
**CORE: Blood Pressure**				
87	Interviewer ID		□□□□□□□	M8
88	Device ID for blood pressure		□□□□□	M9
89	Cuff size used	Small	1	M10
		Medium	2	
		Large	3	
90	Reading 1	Systolic (mmHg)	□□□□□□□	M11a
		Diastolic (mmHg)	□□□□□□□	M11b
91	Reading 2	Systolic (mmHg)	□□□□□□□	M12a
		Diastolic (mmHg)	□□□□□□□	M12b
92	Reading 3	Systolic (mmHg)	□□□□□□□	M13a
		Diastolic (mmHg)	□□□□□□□	M13b
93	During the past two weeks, have you been treated for raised blood pressure with drugs (medication) prescribed by a doctor or other health worker?	Yes	1	M14
		No	2	

**EXPANDED: Hip Circumference and Heart Rate**				
94	Hip circumference	in Centimeters (cm)	□□□□□□□.□□□	M15
95	Heart Rate			
	Reading 1	Beats per minute	□□□□□□□	M16a
	Reading 2	Beats per minute	□□□□□□□	M16b
	Reading 3	Beats per minute	□□□□□□□	M16c

Step 3 Biochemical Measurements

**CORE: Blood Glucose**			
**Question**		**Response**	**Code**
96	During the past 12 hours have you had anything to eat or drink, other than water?	Yes 1<br>No 2	B1
97	Technician ID	□□□□□□□	B2
98	Device ID	□□□□□	B3
99	Time of day blood specimen taken (24 hour clock)	Hours : minutes □□□□□: □□□□□<br>hrs mins	B4
100	Fasting blood glucose<br>*Choose accordingly: mmol/l or mg/dl*	mmol/l □□□□□. □□□□□<br>mg/dl □□□□□□□.□□□	B5
101	Today, have you taken insulin or other drugs (medication) that have been prescribed by a doctor or other health worker for raised blood glucose?	Yes 1<br>No 2	B6
**CORE: Blood Lipids**			
102	Device ID	□□□□□	B7
103	Total cholesterol<br>*Choose accordingly: mmol/l or mg/dl*	mmol/l □□□□□. □□□□□<br>mg/dl □□□□□□□.□□□	B8
104	During the past two weeks, have you been treated for raised cholesterol with drugs (medication) prescribed by a doctor or other health worker?	Yes 1<br>No 2	B9

**EXPANDED: Triglycerides and HDL Cholesterol**			
105	Triglycerides<br>*Choose accordingly: mmol/l or mg/dl*	mmol/l □□□□□. □□□□□<br>mg/dl □□□□□□□.□□□	B10
106	HDL Cholesterol<br>*Choose accordingly: mmol/l or mg/dl*	mmol/l □□□. □□□□□<br>mg/dl □□□□□□□.□□□	B11

**EXPANDED: High Sensitivity CRP**			
107	Hs CRP<br>*Choose accordingly: mmol/l or mg/dl*	mmol/l □□□□□. □□□□□<br>mg/dl □□□□□□□.□□□	B12

World Health Organization
